# S77. JUMPING TO CONCLUSIONS AND FACIAL EMOTION RECOGNITION IMPAIRMENT IN FIRST EPISODE PSYCHOSIS ACROSS EUROPE

**DOI:** 10.1093/schbul/sby018.864

**Published:** 2018-04-01

**Authors:** Giada Tripoli, Diego Quattrone, Charlotte Gayer-Anderson, Victoria Rodriguez, Natashia Benzian-Olsson, Laura Ferraro, Caterina La Cascia, Crocettarachele Sartorio, Lucia Sideli, Fabio Seminerio, Daniele La Barbera, Craig Morgan, Pak Sham, Marta Di Forti, Robin Murray

**Affiliations:** 1 Institute of Psychiatry, Psychology & Neuroscience, King’s College London; 2 University of Palermo; 3 The University of Hong Kong

## Abstract

**Background:**

Jumping to conclusions (JTC) is a well-established reasoning and data gathering bias found in patients with psychosis even at illness onset (First Episode Psychosis, FEP). Preliminary work in this field focused primarily on the association with delusions, although jumping to conclusions has also been found in non-deluded schizophrenia patients after remission, and in individual with at risk mental state.

Moreover, psychotic patients tend to show impairments in social cognition, struggling in identifying, processing and interpreting social clues. Deficits in facial emotion recognition (FER) – a key component of the construct – represent a well-replicated finding in schizophrenia. Furthermore, deficits in global facial affect recognition have been found in FEP with the same severity as at further stages, especially for anger recognition. The present study aims to measure JTC and FER bias in a sample of FEP recruited across 5 European countries, compared with healthy controls.

**Methods:**

Data on JTC (Beads task 60:40), FER (Degraded Facial Recognition task – DFAR) and socio-demographics have been analysed in a sample of 643 FEP and 1019 population controls recruited as part as the EU-GEI study across UK, Netherlands, France, Spain, and Italy.

IQ scores were used to exclude cases and controls with current IQ<70 (N=171) from JTC analysis and a score <41 (N=384) on the Benton Facial Recognition test for the analysis on DFAR. Logistic regression model was applied to predict case/control status using 1) JTC and 2) DFAR as predictive variables controlling for age, gender and country.

**Results:**

We showed that the presence of JTC bias varies across different countries both in cases (χ2=23.77 p<0.001) and controls groups (χ2=14.01 p=0.007).

Logistic regression analyses revealed JTC to be a significant predictor of case/control status (Adj OR=1.88 CI 95%=1.43–2.29 p<0.001).

As well as JTC, FER differed over Europe in both groups (FEP, total: F=17.37, p<0.001; neutral: F=12.4, p<0.001; happy: F=25.62, p<0.001; frightened: F=8.78, p<0.001; angry: F=5.48, p<0.001. Controls, total: F=23.06, p<0.001; neutral: F=21.72, p<0.001; happy: F=21.74, p<0.001; frightened: F=14.14, p<0.001; angry: F=12.49, p<0.001).

Logistic regression analyses revealed all DFAR scores, except for happy emotions, to be negatively associated with case/control status (total: B=-.0182 p=0.001; neutral: B=-.054 p=0.003; happy: B=-.0196 p=0.2; frightened: B= -.065 p<0.001; angry: B=-.030 p=0.04).

**Discussion:**

This study supports the evidence that 1) FEP patients are more likely to present JTC and FER impairments than controls; 2) cognition and social cognition might represent transcultural features of psychotic disorders.

